# The Impact of Kundalini Yoga on Cognitive Function and Memory: A Systematic Review of Randomized Controlled Trials

**DOI:** 10.7759/cureus.63161

**Published:** 2024-06-25

**Authors:** Selvaraj Giridharan, Nagaraj V Kumar, Rajanee Bhana

**Affiliations:** 1 Oncology, Tawam Hospials, Al Ain, ARE; 2 Emergency Medicine, Tawam Hospital, Al Ain, ARE; 3 Oncology, University Hospitals North Midlands, Stoke-on-Trent, GBR

**Keywords:** hippocampal volume, dementia, memory impairment, cognitive function, kundalini yoga

## Abstract

Cognitive decline and dementia are significant public health challenges influenced by various modifiable and non-modifiable risk factors. Kundalini yoga (KY) has emerged as a promising non-pharmacological intervention to enhance cognitive function and memory in older adults at risk of cognitive decline. This systematic review aims to evaluate the effects of KY on cognitive function, memory impairment, and related neurobiological and psychological outcomes in older adults.

A comprehensive literature search was conducted across PubMed, MEDLINE, Scopus, Web of Science, and the Cochrane Library, covering studies published from January 2000 to December 2023. Randomised controlled trials (RCTs) were included to compare KY with other cognitive enhancement strategies, such as memory enhancement training (MET) and psychoeducation.

Five RCTs with 215 participants met the inclusion criteria. The studies varied in sample size (11 to 81 participants) and duration (12 to 24 weeks). The participants were older adults (≥55 years) with mild cognitive impairment (MCI) or subjective cognitive decline (SCD). The interventions compared KY with MET or psychoeducation. KY consistently improved memory performance and executive function. Significant mood enhancements, increased hippocampal volume, and better neural connectivity were observed. KY also reduced pro-inflammatory cytokines and altered ageing-related gene expression, demonstrating both cognitive and neurobiological benefits.

KY appears to be a promising intervention for enhancing cognitive function, mood, and neurobiological health in older adults at risk of cognitive decline and dementia. While further research with more extensive, well-designed RCTs is needed to confirm these findings and optimise intervention strategies, the existing evidence supports the integration of KY into cognitive health programmes. Practitioners should ensure proper training and gradual progression to maximise benefits and minimise risks.

## Introduction and background

Cognitive decline and dementia represent significant health challenges in the general populace, influenced by a range of risk factors. While ageing stands as a primary, unalterable risk factor for dementia [1], several modifiable risk factors, such as diabetes mellitus, hypertension, hypercholesterolemia, hypothyroidism, alcohol intake, and depression, also exert a critical influence on cognitive decline [2]. Notably, individuals with a history of stroke face a heightened risk of developing dementia compared to those without such a history [3]. Furthermore, lifestyle factors, including social activity and diet, are intricately linked to cognitive health, with adherence to a Mediterranean diet associated with a reduced risk of dementia and cognitive decline [4].

Various strategies can be utilised to combat cognitive decline, including mind-body exercises like yoga. Research indicates that yoga, including Kundalini yoga (KY), may positively impact cognitive functions, particularly attention and verbal memory [5]. Yoga interventions can induce neurophysiological changes and stress regulation mechanisms that positively influence cognitive health [6].

KY has been associated with enhancing memory and cognitive function in individuals with mild cognitive impairment and dementia [7]. Additionally, KY has been shown to improve emotional well-being, social perception, and overall quality of life [8]. Furthermore, yoga, including KY, has been linked to neuroprotective effects that may prevent neurodegenerative changes and cognitive decline over short periods [9]. Regular yoga practice has been connected to positive effects on cellular ageing, mobility, balance, mental health, and the prevention of cognitive decline, all vital for older adults [10].

KY, as taught by Yogi Bhajan, encompasses six major components: tuning in with a mantra pranayam (breathing exercises) or warm-up, kriya (a set of exercises), relaxation, meditation, and closing with a blessing song. Kriyas, which can range from simple sequences to vigorous exercises, aim to strengthen the nervous and endocrine systems. Pranayam practices include various breathing techniques such as Breath of Fire and alternate nostril breathing. Meditations often involve movement, mantra, eye focus (drishti), mudra (hand position), and asana (body posture). A typical session lasts 60-90 minutes, including a warm-up, kriya, relaxation, and meditation [11].

The practice of KY involves a range of techniques, including specific postures (asanas), breathing exercises (pranayama), meditation, and chanting (mantras). These techniques help balance the body's energy, reduce stress, and enhance mental clarity. KY can be tailored to individual needs and adjusted to different levels of physical fitness and experience. KY is believed to modulate the autonomic nervous system through practices such as pranayama, which induces relaxation and reduces stress [12]. Meditation techniques like Kirtan Kriya may impact cognitive decline and mental well-being [13].

Research has indicated that KY can enhance cognitive function, resilience, neurochemistry, and neuroplasticity [14]. Additionally, it has been shown to improve emotional well-being, social perception, and overall quality of life. For instance, studies have demonstrated that KY can increase hippocampal volume, improve neural connectivity, and reduce inflammatory markers, which are essential in cognitive decline and dementia progression [15, 16]. KY's focus on spiritual practice, mental engagement, and specific breathing techniques distinguishes it from other forms of yoga, which often emphasise physical postures.

The impact of KY on cognitive function in older adults has been explored in numerous randomised controlled trials (RCTs), comparing it with other cognitive enhancement strategies such as memory enhancement training (MET) and psychoeducation. These studies provide valuable insights into the potential benefits of KY and its mechanisms of action. However, the quality and consistency of the evidence vary, making it necessary to conduct a systematic review to synthesise findings and assess the overall effectiveness of KY in enhancing cognitive health. This review aims to synthesise existing evidence, assess neurobiological changes, evaluate psychological benefits, identify and address bias, and provide clinical recommendations. By conducting a systematic review of the available evidence, this study aims to clarify the role of KY as a non-pharmacological intervention for cognitive decline and dementia, providing a comprehensive understanding of its benefits and limitations. Additionally, the review will emphasise areas for future research, highlighting the need for larger, well-designed RCTs to confirm and extend the findings.

## Review

Methods

A comprehensive literature search was carried out to identify RCTs evaluating the effects of KY on cognitive function and memory impairment. The search included PubMed, MEDLINE, Web of Science, Scopus, and the Cochrane Library, covering studies published from January 2000 to December 2023. Search terms included "Kundalini yoga," "cognitive function," "memory impairment," "dementia," "Alzheimer's disease," and "randomised controlled trial."

Inclusion and Exclusion Criteria

The study aims to investigate the effects of KY on older adults with mild cognitive impairment (MCI), subjective cognitive decline (SCD), or those at risk for dementia. Diagnoses of MCI and SCD were based on standard clinical criteria, including the Petersen criteria for MCI and self-reported cognitive concerns without significant impairment in daily functioning for SCD. The inclusion criteria require participants to be aged 50 or above, and the intervention involves KY, which includes breathing, meditation, chanting, and physical postures. The comparison groups include MET or other non-pharmacological interventions. The outcomes measured include cognitive measures (e.g., memory, executive function), mood assessments (e.g., depression, anxiety), and neurobiological outcomes (e.g., hippocampal volume, inflammatory markers). Only RCTs are considered. Studies that do not meet these criteria, include participants with severe cognitive impairment or other neurological conditions not specified as dementia or MCI, or do not use standardised cognitive or neurobiological assessments are excluded. Only articles published in English were included. The data extracted include study characteristics, intervention details, comparison groups, and outcome measures. The results focus on primary and secondary outcomes, statistical significance, and effect sizes.

Bias Assessment

The risk of bias in the included studies was assessed using the Cochrane Risk of Bias Tool for Randomised Controlled Trials (RoB 2) [17]. This tool evaluates bias across five domains: the randomisation process, deviations from intended interventions, missing outcome data, measurement of the outcome, and selection of the reported result. Each domain was rated as low, high, or having some concerns. Discrepancies between reviewers were resolved through discussion and consensus.

Data Synthesis and Analysis

The results were synthesised qualitatively, focusing on the effects of KY on cognitive function, mood, and neurobiological outcomes. Heterogeneity among studies was assessed, and publication bias was evaluated.

This review adheres to the PRISMA (Preferred Reporting Items for Systematic Reviews and Meta-Analyses) guidelines for conducting and reporting systematic reviews [18].

Results

**Figure 1:**
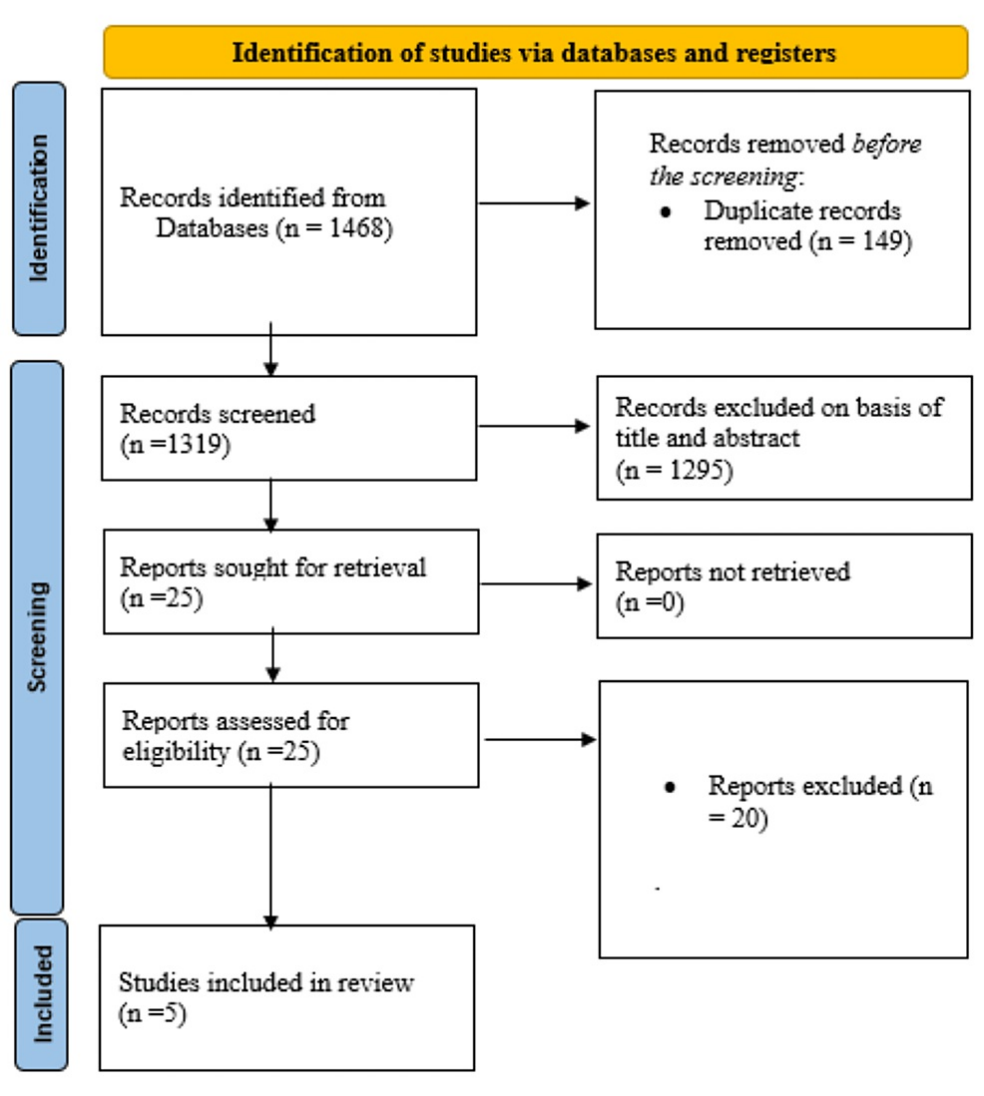
Summarized search strategy (Preferred Reporting Items for Systematic Reviews and Meta-Analyses flow diagram)

The search process identified 1468 items from all the databases examined, as depicted in Figure 1.

Before the screening phase, 149 duplicate records were eliminated, resulting in 1319 records for the initial screening. Subsequently, 1294 records were excluded based on their titles and abstracts, leaving 25 reports. During the subsequent stage, additional papers were excluded based on predefined criteria that did not feature KY as the primary intervention. Five RCTs meeting all inclusion criteria were deemed suitable for review, involving 215 participants [19-23]. The summary characteristics of the studies are outlined in Table 1.

**Table 1:** Summary characteristics of studies included in the review KY: Kundalini yoga, MET: memory enhancement training, SCD: subjective cognitive decline, MCI: mild cognitive impairment, CVRF: cerebrovascular risk factors.

Study	Sample Size	Participant Demographics	Intervention	Duration	Outcomes Measured
Eyre et al. [19]	81	Older adults (≥55 years) with MCI	KY vs. MET	12 weeks	Memory, executive function, mood (depression, resilience)
Grzenda et al. [20]	79	Older women with SCD and CVRFs	KY vs. MET	12 weeks	Memory, subjective cognitive impairment, inflammatory markers
Ibrahim et al. [21]	11	Healthy, meditation-naïve older adults	KY vs. psychoeducation	12 weeks	Hippocampal volume
Kilpatrick et al. [22]	22	Older women with SCD and CVRFs	KY vs. MET	12 weeks	Hippocampal connectivity, stress, memory
Krause-Sorio et al. [23]	22	Older women with SCD and CVRFs	KY vs. MET	12 weeks	Gray matter volume, hippocampal volume

The studies varied in sample size, ranging from 11 to 81 participants, and the intervention durations spanned from 12 to 24 weeks. The participants were older adults (≥55 years) with MCI or SCD, and the interventions compared KY with MET or psychoeducation.

Risk of Bias Assessment

Eyre et al. [19] consistently showed low risk across all domains, which boosts confidence in the study's findings. Grzenda et al. [20] and the smaller studies [21-23] exhibited higher risks due to small sample sizes and differential dropout rates between intervention groups. Table 2 summarises the risk of bias assessment.

**Table 2:** Risk of bias summary

Study	Randomization Process	Deviations From Intended Interventions	Missing Outcome Data	Measurement of the Outcome	Selection of the Reported Result	Overall Risk of Bias
Eyre et al. [18]	Low	Low	Low	Low	Low	Low
Grzenda et al. [19]	High	Low	High	Low	Low	High
Ibrahim et al. [20]	High	Low	Low	Low	Low	High
Kilpatrick et al. [21]	High	Low	Low	Low	Low	High
Krause-Sorio et al. [22]	High	Low	Low	Low	Low	High

Quality of Evidence

The quality of evidence for each outcome was assessed using the GRADE framework. Table 3 summarises the GRADE assessment for the key outcomes evaluated in this review.

**Table 3:** GRADE assessment summary for key outcomes in the included studies

Outcome	Number of Studies	Risk of Bias	Inconsistency	Indirectness	Imprecision	Publication Bias	Overall Quality
Memory improvement	5	High	Low	Low	Low	Low	Moderate
Executive function	3	High	Low	Low	Low	Low	Moderate
Mood (depression, resilience)	3	Low	Low	Low	Low	Low	High
Hippocampal volume and connectivity	4	High	Low	Low	Low	Low	Moderate
Inflammatory markers	2	High	Low	Low	High	Low	Low

Cognitive Outcomes

Eyre et al. demonstrated significant improvements in memory performance and executive function in older adults with MCI in the KY group [19]. Grzenda et al. found that participants in the KY group, particularly older women with SCD and cerebrovascular risk factors, showed significant improvements in subjective cognitive impairment [20]. Ibrahim et al. reported a significant increase in right hippocampal volume in the KY group, comprising 11 healthy, meditation-naïve older adults [21]. Kilpatrick et al. and Krause-Sorio et al. showed that KY increased hippocampal connectivity and volume in older women with SCD and cerebrovascular risk factors [22,23].

Mood and Psychological Outcomes

Eyre et al. and Grzenda et al. reported significant improvements in depressive symptoms and resilience in the KY groups compared to the control groups [19,20]. These mood improvements were sustained over the 24-week follow-up period. Krause-Sorio et al. observed no significant changes in anxiety, depression, or stress levels, likely due to the relatively healthy baseline mental status of participants [22]. However, the observed neuroprotective effects may indirectly contribute to improved psychological well-being.

Neurobiological Outcomes

Grzenda et al. identified that KY uniquely altered ageing-associated gene expression signatures, including reductions in pro-inflammatory cytokines [20]. These changes were not observed in the MET group, suggesting that KY may provide anti-inflammatory and anti-ageing benefits at a molecular level. Ibrahim et al. and Kilpatrick et al. reported significant increases in hippocampal volume and improved connectivity in KY participants [21,22]. These neurobiological changes underpin the cognitive improvements observed in KY groups, highlighting the practice's potential for enhancing brain health. Krause-Sorio et al. observed neuroprotective effects, including the prevention of grey matter atrophy in key brain regions associated with cognitive function. These findings support the hypothesis that KY may mitigate neurodegenerative changes.

Discussion

The systematic review reveals that KY confers significant benefits for cognitive function, mood, and neurobiological health in older adults at risk for cognitive decline and dementia. Across five RCTs, KY consistently demonstrated improvements in memory and executive function, reductions in depressive symptoms, and enhanced resilience. Neurobiological assessments indicated increased hippocampal volume, improved connectivity, and favourable inflammatory and ageing-related biomarker alterations.

These findings substantiate prior research emphasising the cognitive and psychological advantages of mind-body interventions such as yoga. Notably, KY's emphasis on meditation, breathwork, and spiritual practice may confer distinctive benefits compared to other forms of physical exercise and cognitive training. The documented enhancements in hippocampal volume and connectivity across multiple studies support the proposition that KY may elicit neuroplastic changes, potentially slowing age-related cognitive decline.

While our review focuses specifically on the cognitive, mood, and neurobiological benefits of KY in older adults with MCI or SCD, other research has examined a broader range of yoga practices and their effects on cognitive function in various populations. For instance, Karamacoska et al. reviewed the health effects of yoga for individuals with MCI and dementia, noting improved cognition, mood, and balance​​ [24]. They also highlighted the high risk of bias and the need for more rigorous studies. Our findings suggest that KY can significantly enhance cognitive function and mood in at-risk older adults. However, the unique emphasis on spiritual and meditative practices in KY may provide additional benefits not captured in studies of other yoga types. Our review's focus on neurobiological outcomes, such as hippocampal volume and connectivity, provides further evidence of KY's potential to induce neuroplastic changes and reduce neuroinflammation.

Eilat-Adar et al. conducted a systematic review evaluating different yoga interventions and their effects on cognitive functions in healthy older adults [25]. Some studies found that yoga improved working memory, executive functions, visual memory, and processing speed. However, their review included diverse yoga types, such as Hatha, Trataka, Iyengar, and Himalayan Siddha, which varied significantly in intervention length and frequency​. The studies reviewed by Eilat-Adar et al. also involved larger sample sizes than our focus on smaller RCTs. While their findings support the cognitive benefits of yoga, our review highlights that KY, specifically, may offer unique neuroprotective benefits. 

Similarly, Hoy et al.'s systematic review of yoga-based interventions for cognitive function in healthy older adults found that four out of six studies reported significant cognitive improvements, including gross memory and executive functions [26]. However, they noted a high risk of bias and variability in intervention characteristics, which complicates the generalisation of results. Hoy et al. emphasised the need for adequately powered RCTs with standardised protocols, a recommendation that our review echoes. Unlike Hoy et al., our review focuses exclusively on KY, demonstrating consistent cognitive and neurobiological benefits across studies despite smaller sample sizes and varied methodologies.

The cognitive advantages associated with KY are multifaceted. Increased hippocampal volume and connectivity suggest that KY enhances neuroplasticity, which is critical for memory formation and cognitive function [21-23]. Furthermore, reductions in pro-inflammatory cytokines and alterations in gene expression related to ageing highlight KY's potential to modulate the immune response, thereby reducing neuroinflammation and mitigating cognitive decline [20]. KY's emphasis on breathwork and meditation may also mitigate stress, a known negative factor for cognitive health. The observed enhancements in mood and resilience further substantiate this mechanism. Additionally, yoga practices are known to increase brain-derived neurotrophic factor (BDNF) levels in the brain, which can further contribute to cognitive improvements and overall brain health [27].

The practice of KY offers significant benefits but is not without associated risks. Physical side effects, such as headaches, back pain, and fatigue, as well as psychological effects, including emotional turbulence and mood swings, have been reported. It is crucial to emphasise the importance of engaging in KY under the guidance of experienced instructors to ensure safe and effective practice [28].

The study findings indicate that KY could be integrated into cognitive health interventions for older adults, particularly those at risk for Alzheimer's disease and other forms of dementia. Practitioners should be mindful of potential side effects and take measures to mitigate these risks through proper training and gradual progression. Integrating KY with traditional memory enhancement strategies may provide comprehensive benefits, targeting multiple aspects of brain health and cognitive function.

However, it is essential to note this review's limitations. The included studies varied in sample size and methodological quality, with some exhibiting a high risk of bias due to small sample sizes and differential dropout rates. Factors contributing to this high risk include small sample sizes, differential dropout rates between intervention groups, and potential inconsistencies in intervention protocols. These limitations suggest that while the findings are promising, they should be interpreted with caution. Furthermore, the heterogeneity in intervention protocols and outcome measures complicates direct study comparisons. Future research should standardise KY interventions and employ more extensive, more diverse samples to enhance the generalisability of findings.

Future research directions include the need for more extended follow-up periods to assess the sustained effects of KY on cognitive function and neurobiological health. Additionally, further research should delve into the underlying mechanisms of KY's cognitive benefits, including its impact on neuroplasticity, inflammation, and stress pathways. Studies should also include more diverse populations to determine the applicability of findings across different demographic groups. Incorporating placebo or control arms in trials will provide more robust comparisons and help isolate the specific effects of KY.

## Conclusions

KY appears to be a promising intervention for improving cognitive function, mood, and neurobiological health in older adults at risk for cognitive decline and dementia. While further research is needed to confirm these findings and optimise intervention strategies, the existing evidence supports the integration of KY into cognitive health programs. Practitioners should approach KY cautiously, ensuring proper training and gradual progression to maximise benefits and minimise risks.
